# IgG4-related digestive diseases: diagnosis and treatment

**DOI:** 10.3389/fimmu.2023.1278332

**Published:** 2023-10-05

**Authors:** Siyu Wu, Haiqiang Wang

**Affiliations:** ^1^ Graduate School, Heilongjiang University of Chinese Medicine, Harbin, China; ^2^ Department of Internal Medicine, First Affiliated Hospital, Heilongjiang University of Chinese Medicine, Harbin, China

**Keywords:** IgG4, digestive disease, autoimmune reaction, diagnosis, treatment

## Abstract

IgG4-related digestive diseases encompass a group of chronic inflammatory disorders characterized by autoimmune reactions and fibrosis affecting multiple digestive organs. These diseases are identified by elevated serum levels of IgG4 and the presence of IgG4-positive plasma cell infiltration in the affected sites, along with storiform fibrosis, obliterative phlebitis, and eosinophilic infiltration. Although extensive research has been conducted, a comprehensive understanding of these conditions remains elusive. Current clinical diagnosis often relies on the application of integrated diagnostic criteria for IgG4-related diseases, combined with specific organ involvement criteria. Distinguishing them from malignancies poses considerable challenges. Moreover, further investigations are required to elucidate the underlying pathogenic mechanisms and explore potential therapeutic interventions. This review provides a systematic classification of IgG4-related digestive diseases while discussing their diagnostic strategies, clinical presentations, and treatment modalities. The comprehensive insights shared herein aim to guide clinicians in their practice and contribute to the advancement of knowledge in this field.

## 1 Introduction

Immunoglobulin G4-related disease (IgG4-RD) is a recently recognized and extensively studied immune-mediated disorder that manifests as fibroinflammatory lesions affecting multiple organs throughout the body, often characterized by the formation of pseudo-tumorous masses and tissue sclerosis. In 2012, the international medical community standardized the nomenclature for this class of diseases as IgG4-RD (1).

**Figure 1:**
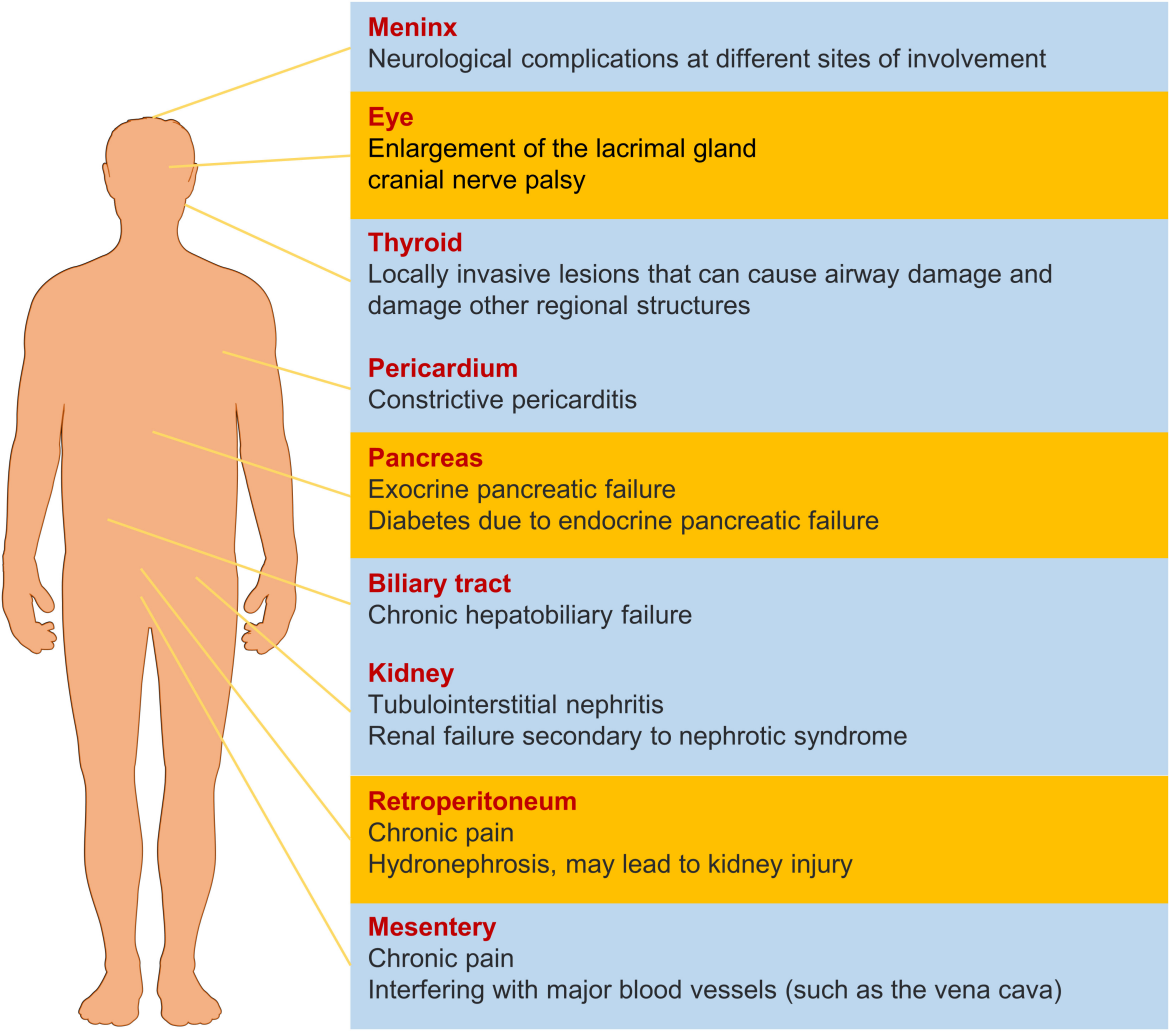
The effect of IgG4-RD in different organs. The yellow items represent a higher probability of morbidity.

IgG4-RD displays unique clinical features and pathological manifestations, predominantly affecting middle-aged and elderly men (2). The disease typically presents with a subacute onset and can involve virtually any organ (**Figure 1**), including the pancreas, biliary system, salivary glands, orbital tissues, kidneys, lungs, lymph nodes, meninges, aorta, breasts, prostate, thyroid, pericardium, and skin (3). Depending on the site of involvement, IgG4-RD can be classified into four phenotypes, with hepatic, pancreatic, and biliary involvement accounting for 31%, retroperitoneal fibrosis and head and neck lesions each accounting for 24%, and Mikulicz’s syndrome without systemic involvement comprising 22% of cases (4–6). Thus, the digestive system is the most commonly affected site of IgG4-RD (7).

**Figure 2:**
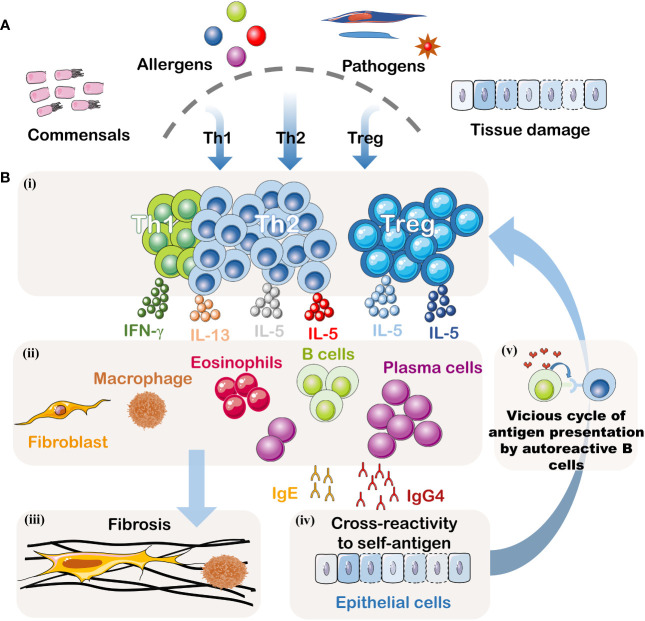
Pathogenesis of IgG4-DSD. **(A)** Potential Triggering Factors: Although the initial event is unknown, this disease may result from abnormal immune responses to symbiotic microorganisms, food and environmental allergens, infectious pathogens, or tissue damage. **(B)**(i) Signals from the innate immune system may dictate the polarization of T helper cells. When inflammation becomes chronic, Treg cells may also be suppressed. **(ii)** Activated T helper cells and Treg cells may create an inflammatory cytokine environment, including IFN-γ, IL-4, IL-10, IL-5, and IL-13. IL-4 and IL-10 may be produced by T follicular helper cells, driving the preferential class switching of auto-reactive B cells to IgG4 and IgE and inducing differentiation and expansion of IgG4+ plasma cells. IL-5, IL-13, and TGF-β may lead to the recruitment of eosinophils and activation of fibroblasts. This cytokine milieu may also promote fibrosis or activate macrophages to produce additional fibrogenic cytokines. IFN-γ may contribute to the activation of inflammatory macrophages driving the fibrotic process. **(iii)** Activated fibroblasts and macrophages produce a dense fibrotic pattern. (iv) Some IgG4 antibodies, possibly accompanied by some IgE antibodies, may cross-react with autoantigens. (v) B cells recognizing autoantigens can present effective antigens derived from homologous autoantigens to auto-reactive T cells, thus establishing a vicious cycle between malignant T lymphocytes and B lymphocytes. These activated auto-reactive T cells may contribute to the formation of germinal centers and recruit increasingly high-affinity auto-reactive B cell clones.

This article provides an overview of IgG4-related digestive system diseases (IgG4-DSD). Currently, the understanding of the pathogenic mechanisms underlying this condition remains limited, with both genetic and environmental factors contributing to its development. The disease’s etiology primarily involves dysregulation of the immune system, encompassing innate and adaptive immunity, as well as interactions between B cells and T cells (**Figure 2**). With a growing emphasis on patient safety in recent years, awareness regarding the diagnosis and treatment of IgG4-DSD has deepened. Therefore, this article classifies IgG4-related digestive system diseases and focuses on their diagnosis and treatment, aiming to provide guidance for clinical practice.

## 2 Common diagnostic features of IgG4-DSD

**Figure 3:**
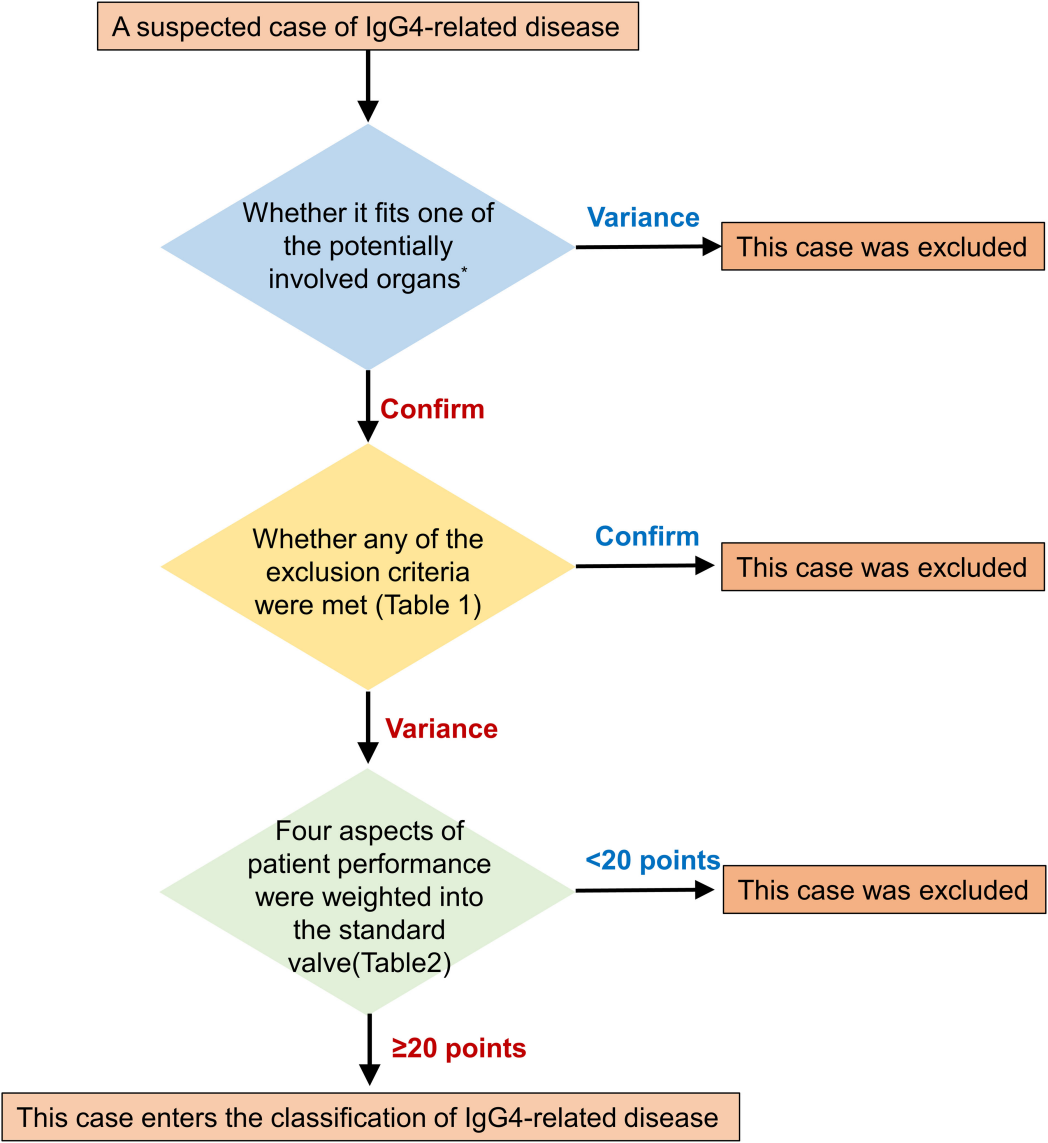
Step-by-step sketch of IgG4-RD classification. The potentially involved organs includes Pancreas, salivary glands, bile ducts, orbit, kidney, lung, aorta, retroperitoneum, dura mater, or thyroid (Riedel's thyroiditis).

IgG4-DSD is a type of IgG4-RD characterized by multi-organ fibroinflammatory involvement. Its clinical presentation is diverse, with a lack of specific biomarkers, imaging findings, and laboratory tests, making diagnosis challenging. Thus, IgG4-DSD is often confused with other diseases and malignancies, leading to misdiagnosis and underdiagnosis. To address this issue, a three-step classification for IgG4-RD was proposed by the European League Against Rheumatism and the American College of Rheumatology in 2019 (**Figure 3** and **Tables 1**, **2**), which can be used for initial categorization (8). The establishment of a methodology involving a 3-step initial classification process, which includes the incorporation of suspected cases, exclusion of similar diseases, classification assessment, and numerical weighting, enables the direct diagnosis of IgG4-RD based on clinical presentation, serology, imaging, and pathology. Biopsy is no longer required, and a significant elevation of IgG4 levels is no longer the sole criterion for diagnosing this condition.

**Table 1:** Exclusion criteria in IgG4-RD classification.

Category	Criterion
Clinic	Fever
	No objective response to glucocorticoids
Serology	No other explanation for leukopenia and thrombocytopenia in serology
	Peripheral eosinophilia was present
	ANCA-positive: a positive ELISA against PR3 or MPO-ANCA
	Ro and La antibodies were positive
	Antibodies against dsDNA, RNP, or Sm were positive
	Other disease-specific autoantibodies
	Cryoglobulinemia
Radiology	Known radiological findings of suspected malignancy or infection have not been fully investigated
	Rapid radiological progress
	Consistent with an abnormal disease of the long bones of Erdheim-Chester
Pathology	Cellular infiltration of an understudied suspected malignancy
	Markers consistent with inflammatory myofibroblastic tumor
	Marked neutrophilic inflammation
	Necrotizing angitis
	Marked necrosis
	Mainly granulomatous inflammation
	Pathological features of macrophage/histiocytic diseases
Known diagnose	Multicentric Castleman's disease
	Crohn's disease or ulcerative colitis (if pancreaticobiliary disease is present)

**Table 2:** Weighting criteria in IgG4-RD classification.

Category	Criterion	Score
Histopathology	No pathological information	0
	dense lymphocytic infiltrate	4
	dense lymphoplasmacytic infiltrate with obliterative phlebitis	6
	Dense lymphocyte infiltration and storiform fibrosis with or without obliterative phlebitis	13
Immunostaining	The lgG4+:IgG+ ratio ranged from 0% to 40% or was indeterminate and the number of IgG4+ cells /hpf ranged from 0 to 9	0
	(1) lgG4+:lgG+ ratio >41% and the number of lgG4+ cells /hpf was 0-9 or indeterminate; (2) lgG4+:lgG+ ratio was 0-40% or indeterminate, and the number of IgG4+ cells /hpf was >10 or indeterminate	7
	(1) The ratio of lgG4+ to lgG+ was 41%-70% and the number of IgG4+ cells /hpf was more than 10; (2) The lgG4+:lgG+ ratio was >71%, and the number of lgG4+ cells /hpf was 10-50	14
	lgG4+:lgG+ ratio >71% and IgG4+ cells /hpf>51	16
Serum IgG4 concentration	Normal or not examined	0
	Between normal values and 2 times normal values	4
	2-5 times the normal value	6
	≥5 times normal value	11
Bilateral lacrimal, parotid, sublingual and submandibular glands	No glandular involvement	0
	A group of glands was involved	6
	Two or more groups of glands were involved	14
Chest	No examination or none of the following occurred	0
	Peribronchovascular and septal thickening	4
	Paravertebral band- like soft tissue in the thorax	10
Pancreas and biliary tree	Not checked or none of the items listed is present	0
	Diffuse pancreas enlargement (loss of lobulations)	8
	Diffuse pancreas enlargement and capsule- like rim with decreased enhancement	11
	Pancreas (either of above) and biliary tree involvement	19
Kidney	Not checked or none of the items listed is present	0
	Hypocomplementemia	6
	Renal pelvis thickening/soft tissue	8
	Bilateral renal cortex low- density areas	10
Retroperitoneum	Not checked or neither of the items listed is present	0
	Diffuse thickening of the abdominal aortic wall	4
	Circumferential or anterolateral soft tissue around the infrarenal aorta or iliac arteries	8

However, due to the significant variations in clinical symptoms among different organ involvements, there is currently no unified diagnostic criteria. The updated comprehensive diagnostic criteria for IgG4-related disease in 2020 can be consulted (9). The following is a summary of the common features of IgG4-DSD.

Firstly, elevated serum and tissue levels of IgG4 are important diagnostic criteria. The current consensus considers a serum IgG4 cutoff value of 1.35 g/L. However, different studies have defined this cutoff value differently. Research has shown that a serum IgG4 level greater than 2.5 g/L helps distinguish primary sclerosing cholangitis (PSC) from IgG4-related sclerosing cholangitis (IgG4-SC), with a sensitivity of 67%-89% and specificity of 95% (10). Some studies have even used a serum IgG4 level greater than 5.6 g/L as the threshold to differentiate IgG4-SC from PSC and bile duct cancer, thereby increasing the specificity and positive predictive value to 100% (11). Furthermore, research has indicated a positive correlation between elevated serum IgG4 levels and the number of affected organs, meaning that the higher the disease severity in patients, the more significant the increase in serum IgG4 levels (12). Secondly, histopathological examination is the “gold standard” for diagnosing IgG4-DSD. Although fine-needle aspiration biopsy usually does not provide sufficient tissue samples for diagnosis, it can be used to rule out lymphoma and other malignancies. Various types of IgG4-DSD share common histopathological features (13), including abundant lymphocyte and plasma cell infiltration, widespread and dense fibrosis (often arranged in a swirling pattern), and obliterative phlebitis. Immunohistochemical staining demonstrates abundant IgG4-positive plasma cell infiltration, typically exceeding 10-50 cells/high-power field. Some studies suggest that a proportion of IgG4-positive plasma cells greater than 50% within the overall IgG-producing plasma cell population is more diagnostically significant. The presence of swirling fibrosis and obliterative phlebitis can enhance the specificity of pathological diagnosis (14).

**Figure 4:**
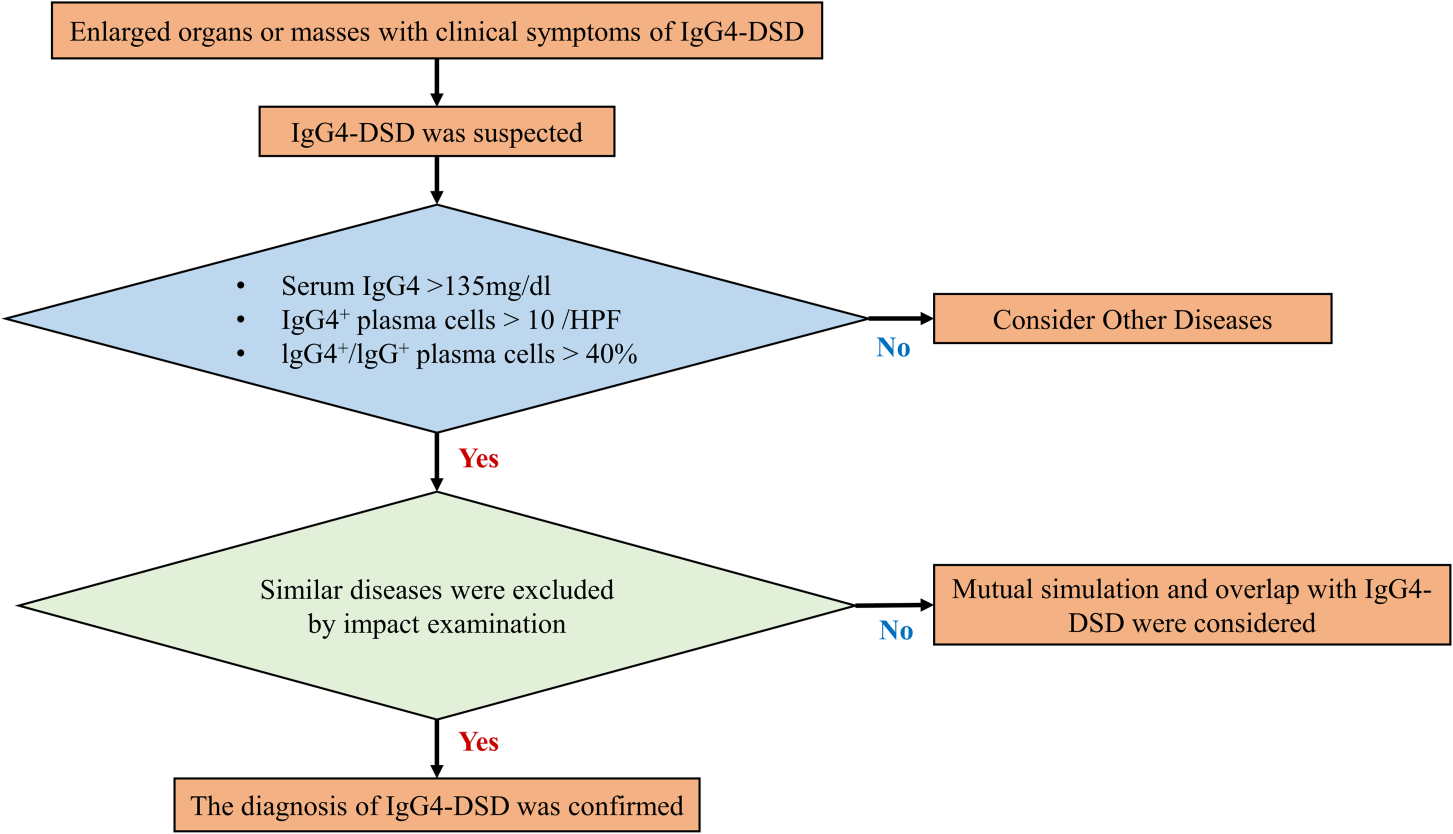
Diagnosis procedures of IgG4-DSD.

Up to this point, the field of IgG4-DSD diagnosis and treatment has been characterized by a dearth of robust medical evidence and a lack of standardization in treatment protocols. The diagnostic procedures have largely relied on the 2011 Japanese comprehensive diagnostic criteria for IgG4-RD (15), as illustrated in **Figure 4**.

## 3 Classification of IgG4-DSD

### 3.1 Autoimmune pancreatitis

Autoimmune pancreatitis (AIP) is a rare disease characterized by an autoimmune reaction and the total prevalence of the disease was 0.0101%, and the annual incidence was 0.0031% (16). According to the guidelines established by the International Association of Pancreatology in 2011, AIP is classified into two types: type 1 and type 2, based on differences in imaging, serology, multisystem involvement, histology, and response to steroid therapy (17). Type 1, also known as lymphoplasmacytic sclerosing pancreatitis, is the most common subtype, accounting for 80-90% of all AIP cases, while type 2 is a rare form characterized by idiopathic duct-centric pancreatitis. In this review, unless otherwise specified, “AIP” refers to IgG4-related type 1 AIP (18).

The onset of AIP is insidious, and typical symptoms include painless obstructive jaundice in elderly males, as well as nonspecific symptoms such as low-grade fever, fatigue, diarrhea, and weight loss. The disease often exhibits recurrent episodes. Acute painful pancreatitis is less common compared to IgG4-negative AIP (19). Research reports indicate that some type 1 AIP patients may present with newly developed hyperglycemia, as AIP can affect both endocrine and exocrine pancreatic function, leading to decreased levels of fecal elastase and increased fasting blood glucose (20).

Currently, the main guidelines used for diagnosing AIP include the International Association of Pancreatology 2011 guidelines (17), Chinese guidelines for the diagnosis and treatment of autoimmune pancreatitis (21), and the Japan Pancreas Society guidelines (22). Despite minor differences in the choice of ancillary tests, stratification of test results, and combination of evidence, these guidelines generally follow similar principles. In addition to considering clinical symptoms, the diagnosis of AIP relies on changes in serum IgG4 levels, imaging examinations such as CT and MRI, as well as endoscopic procedures (e.g., endoscopic retrograde cholangiopancreatography). Although elevated serum IgG4 is a classical serologic marker for type 1 AIP, it is not a necessary condition for diagnosis (17). Some AIP patients may have normal IgG4 levels, and thus normal IgG4 levels do not exclude the presence of AIP. Furthermore, elevated IgG4 levels are not exclusive to AIP, as pancreatic cancer patients may also exhibit elevated levels of serum IgG4. With advancements in laboratory techniques and further research, new biomarkers for AIP continue to emerge. IgG1 is considered to have pathological significance, while IgE, IFN-α, and IL-33 demonstrate similar roles to IgG4 as diagnostic markers for AIP and IgG4-related diseases, particularly in drug efficacy assessment and prediction of recurrence (23). Imaging examinations are pivotal in the diagnostic process of AIP. Characteristic CT/MRI findings typically reveal either focal or diffuse enlargement of the pancreas, although these findings lack specificity. Distinguishing AIP from chronic pancreatitis and pancreatic cancer can be challenging. A notable feature often observed is the loss of lobular structure, resulting in a “sausage-like” appearance of the pancreas. Enhanced CT and MRI scans commonly exhibit delayed enhancement in the hepatic venous phase, with approximately 36% of patients displaying reduced fat space surrounding the pancreas, forming a low-density area with a “halo-sign” appearance, similar to cystic edges (24). Moreover, in patients with type 1 Autoimmune Pancreatitis (AIP), endoscopic ultrasonography (EUS) reveals an absence of stones within the pancreatic duct and no significant dilation of the narrowed upper pancreatic duct. When pancreatic enlargement or mass is present, distinguishing AIP from pancreatic cancer can be challenging. In such cases, further tissue pathology examination through endoscopic ultrasound-guided fine-needle aspiration (EUS-FNA) is necessary. Histopathology is the gold standard for diagnosing type 1 AIP, characterized by notable lymphocyte infiltration and tissue fibrosis (14). Positron emission tomography (PET/CT) often shows abnormally elevated uptake, which can be attributed to an inflammatory response affecting both the pancreas and other organs involved. This imaging modality can also serve as a valuable tool in the differential diagnosis between Autoimmune Pancreatitis (AIP) and pancreatic tumors (25).

**Figure 5:**
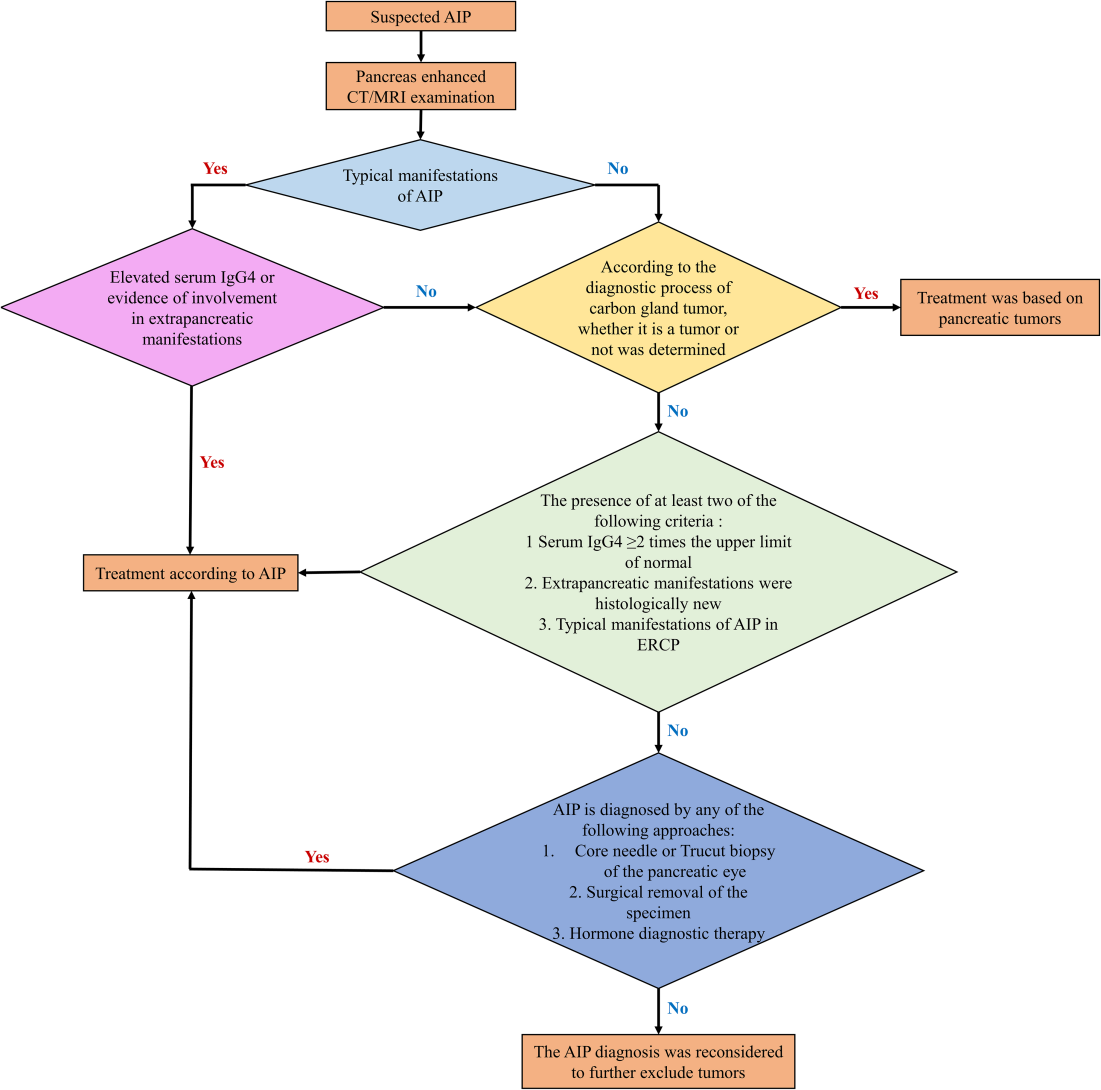
Diagnosis procedures of type 1 AIP.

The diagnostic pathway for AIP is depicted in **Figure 5**. Initially, an imaging examination should be conducted. If the patient exhibits typical imaging findings and there is corroborating evidence from laboratory tests or indications of extra-pancreatic involvement, a diagnosis of AIP can be established. In cases where imaging results are atypical, it is essential to rule out pancreatic cancer. A comprehensive assessment, combining laboratory examinations and histopathological evidence, should guide the judgment. In instances where diagnostic hormone therapy is employed, pancreatic cancer must be definitively excluded, and the treatment regimen should not exceed a duration of 2 weeks. Subsequent imaging reevaluation, demonstrating marked improvements in pancreatic or extrapancreatic lesions, further supports the diagnosis.

### 3.2 IgG4-related sclerosing cholangitis

IgG4-related sclerosing cholangitis (IgG4-SC) is a biliary manifestation belonging to the spectrum of IgG4-related diseases. It is the second most common disease within the IgG4-related diseases, sharing similar clinical and pathological features with AIP. Approximately 88% of IgG4-SC patients also have AIP (26), while involvement of other organs such as salivary glands, retroperitoneum, lacrimal glands, and kidneys is relatively uncommon. Hence, some refer to this condition as AIP-related sclerosing cholangitis (27). IgG4-SC mainly affects middle-aged to elderly males, with a male-to-female ratio of about 4:1 and an average onset age of 67.0 years (28). The main clinical manifestations of this disease include abdominal pain, jaundice, and pruritus. Most patients present with obstructive jaundice, and some may also experience fever, weight loss, and diabetes mellitus, among other abnormalities. A small percentage of patients may develop liver decompensation, presenting with ascites, esophagogastroduodenal varices, gastrointestinal bleeding, and hepatic encephalopathy. About 25% of patients may be asymptomatic (26, 28).

Clinical diagnostic criteria for IgG4-SC include the IgG4-Related Disease consensus diagnostic criteria (9), IgG4-SC clinical diagnostic criteria (29), and HISORT criteria (30). The updated diagnostic criteria introduce a clear demarcation in the diagnostic process between IgG4-SC and IgG4-SC coexisting with AIP, while also enhancing the precision of the differential diagnosis for IgG4-SC subtypes 1-4 (31). Given the striking resemblance in clinical symptoms, serologic markers, and imaging characteristics between IgG4-SC, primary sclerosing cholangitis (PSC), and cholangiocarcinoma (CCA), it becomes imperative to establish a robust and accurate differential diagnosis among these three conditions (refer to **Table 3**).

**Table 3:** Identification of IgG4-SC, PSC, and CCA.

Indication	IgG4-SC	PSC	CCA
Vulnerable population	Elderly person, often associated with AIP	Adolescents or elderly person, often associated with IBD	Elderly person
The level of IgG4 in serum	Rise	Normal	Normal
The effect of glucocorticoid treatment	Effective	Ineffective	Ineffective
Iconography	The thickness of the gallbladder wall was uniform, the inner and outer walls were smooth, the degree of bile duct dilatation was mild, and the bile duct showed segmental stenosis	The bile ducts were banded narrow, beaded, withered dendritic, and diverticuloid eversion	The bile duct wall was asymmetrically thickened, and the bile duct was obviously dilated in a "soft vine" shape, which was easy to invade adjacent blood vessels and lymph node enlargement

In the diagnostic process, elevated levels of serum IgG4 are important evidence for diagnosing IgG4-SC. Approximately 90% of IgG4-SC patients have elevated serum IgG4 levels (32). However, mild elevation of IgG4 can also be observed in PSC, cholangiocarcinoma, and pancreatic cancer patients. Therefore, serum IgG4 levels alone cannot be used as a definitive diagnostic criterion. A multicenter study conducted in Japan suggested that setting the serum IgG4 level at 135 mg/dL could serve as an appropriate diagnostic cutoff value. Additionally, using a cutoff value of 207 mg/dL can accurately differentiate type 3, type 4 IgG4-SC, and cholangiocarcinoma with 100% specificity. Serum laboratory abnormalities in IgG4-SC patients also include elevated bilirubin levels, alkaline phosphatase (ALP), and gamma-glutamyl transferase (GGT) levels. Studies have also shown that plasma M2-pyruvate kinase has potential diagnostic value for IgG4-related diseases, possibly surpassing the value of serum IgG4 (33). In recent years, some scholars have discovered that anti-membrane-protein A11 antibody and anti-laminin-511-E8 antibody are positive in IgG4-SC patients, which can be helpful for diagnosing patients with normal IgG4 levels (34). However, further research is needed to determine whether these antibodies can be used as diagnostic biomarkers.

Imaging plays a pivotal role in the diagnosis of IgG4-SC, which typically exhibits features such as diffuse or segmental stenosis of intra- and extrahepatic bile ducts, thickening of the bile duct wall, and involvement of other organs. Various imaging modalities are employed, including ultrasound endoscopy (EUS), X-ray computed tomography (CT), magnetic resonance imaging (MRI), magnetic resonance cholangiopancreatic water imaging (MRCP), and endoscopic retrograde cholangiopancreatography (ERCP). Among these, EUS and biliary intraluminal ultrasound (IDUS) stand out for their ability to visualize the structural layers of the biliary wall and measure its thickness. EUS is particularly versatile, as it not only assesses wall thickness and identifies intraluminal lesions but also detects pancreatic swelling associated with AIP and allows for tissue biopsy. Typical sonographic features of IgG4-SC include uniformly hypoechoic circumferential thickening of the duct wall and a smooth inner rim (35). Studies have indicated that a wall thickness of 0.8 mm in the non-stenotic segment of the bile duct under IDUS serves as an optimal cutoff value to differentiate IgG4-SC from cholangiocarcinoma, boasting a sensitivity and specificity of over 90% (36). MRI, when evaluating wall thickening, surpasses CT examination, showcasing equal or low signal intensity in T2-weighted images (T2WI), high signal intensity in diffusion-weighted imaging (DWI), and gradual enhancement with a smooth edge and clear border (37). ERCP and MRCP excel in lesion localization and identification, with MRCP offering additional benefits in lesion characterization. These techniques are instrumental in localizing lesions and delineating bile duct morphology. Depending on the location of bile duct stenosis, this condition can be classified into four types, aiding in the differentiation of IgG4-SC from PSC, cholangiocarcinoma (CC), and other disorders (38). In comparison to MRCP, ERCP boasts superior spatial resolution and is considered the “gold standard” for PSC diagnosis, making it an ideal method for distinguishing IgG4-SC from PSC. In ERCP, IgG4-SC typically presents with segmental stenosis of the lower common bile duct, whereas PSC exhibits extensive “bead-like” and “withered twig-like” bile duct stenosis.

Histopathologically, IgG4-SC is characterized by abundant lymphocyte and plasma cell infiltration, IgG4-positive plasma cell infiltration, storiform fibrosis, and obliterative phlebitis, often accompanied by eosinophil infiltration. Lesions usually originate from the mucosal layer of the bile duct wall and gradually extend outwardly, involving not only the strictured segment but also the dilated segments, while the biliary epithelium often remains unaffected (39). Immunohistochemical staining reveals that the presence of more than 10 IgG4-positive cells per high-power field can be used as a criterion for histopathological diagnosis. However, it is not an absolute indicator for diagnosing IgG4-SC, as IgG4-positive plasma cell infiltration is also commonly observed in non-specific inflammation at different sites.

### 3.3 IgG4-related sialadenitis

Among IgG4-DSD, apart from the pancreas and biliary tract, the salivary glands are the third most commonly affected organs and tissues. IgG4-related salivitis mainly includes IgG4-related Mikulicz Diseases (IgG4-MD) and IgG4-related Chronic Sclerosing Sialadentis (IgG4-CSS) (40).

The current diagnostic criteria for IgG4-related salivary gland disease are summarized as follows, taking into account findings from both domestic and international research (41):

1. Clinical Evaluation: Persistent enlargement of the submandibular gland or parotid gland should be observed through clinical examination and auxiliary tests.
2. Laboratory Tests: Elevated levels of serum IgG and IgG4 concentrations (>1.35g/L) should be detected in laboratory tests.
3. Histopathology: Histopathological examination is pivotal. Immunohistochemical results should be interpreted, focusing on positive cells localized within the cytoplasm of plasma cells when viewed under a microscope. Specifically, three areas with high-density positive cells should be selected, and the total count of IgG4-positive and IgG-positive cells should be determined in these three high-power fields. The average count of IgG4-positive and IgG-positive cells in each high-power field should then be calculated. A diagnosis is established if the number of IgG4-positive plasma cells per high-power field is ≥50. In biopsy cases, the criteria may be ≥10 IgG4-positive plasma cells per high-power field, and the ratio of IgG4-positive to IgG-positive plasma cells should be ≥40%.

Based on the degree of fulfillment of the above three diagnostic criteria, the diagnosis is categorized as follows:

- Definitive Diagnosis: Positive for criteria 1, 2, and 3.
- Likely Diagnosis: Positive for criteria 1 and 3.
- Probable Diagnosis: Positive for criteria 1 and 2.

IgG4-CSS and IgG4-MD exhibit distinct clinical manifestations and auxiliary examination findings, which are described as follows.

#### 3.3.1 IgG4-CSS

IgG4-CSS was first discovered and described by the renowned German physician Küttner in 1896. Seifert et al. renamed Küttner’s tumor as Chronic Sclerosing Sialadenitis (CSS) (42). In 2011, Geyer et al. proposed to unify salivary gland lesions of CSS as IgG4-CSS (40), the pathogenesis of which is unclear and may be related to genetic predisposition (43)and abnormal immune response (44).

IgG4-CSS predominantly affects middle-aged men and typically exhibits a protracted disease course. This condition primarily manifests as symmetrical enlargement of the submandibular gland, characterized by a constrained hard texture of the swelling. In some cases, the swelling may present nodular characteristics. Notably, the affected area typically maintains a normal coloration, and the borders of the swelling tend to be well-defined. Additionally, patients may experience mild to moderate dryness in the mouth as a concurrent symptom. Furthermore, IgG4-CSS can extend beyond the submandibular gland, involving other salivary glands such as the parotid gland and the sublingual gland. On rare occasions, this condition can also affect the lacrimal gland (45). Approximately 25% of patients with CSS exhibit systemic symptoms associated with IgG4-related diseases (46, 47). Owing to the persistent enlargement and sclerotic alterations observed in the submandibular gland, IgG4-CSS can often be mistaken for chronic obstructive submandibular inflammation or even neoplastic growth in the submandibular region. Consequently, it is not uncommon for clinicians to opt for surgical removal when confronted with suspected submandibular region tumors. Therefore, meticulous and thorough clinical differential diagnosis is imperative in these cases. When deemed necessary, differentiating the submandibular gland through incisional or excisional biopsy, among other diagnostic techniques, is a viable approach.

Generally, laboratory investigations of CSS reveal elevated levels of IgG4. However, in cases of antigen excess during testing, a double band effect may occur, leading to normal IgG4 levels (48, 49). Furthermore, increased levels of eosinophils and antinuclear antibodies can be detected in peripheral blood, although antibodies against Sjögren syndrome (SS) A and B are negative. Some patients may also display hypocomplementemia (50). CT scans typically show diffuse or nodular increased glandular density with well-defined boundaries. The distinctive histological features of CSS are crucial for differentiating it from tumors. These features include cellular fibrous tissue proliferation and the formation of lymphoid follicles with clearly irregular germinal centers. The main diagnostic criterion is the substantial infiltration of IgG4-positive plasma cells. Studies of immunohistochemical manifestations against CSS showed that the lymphocytes infiltrating the diseased tissues were predominantly T lymphocytes, followed by B lymphocytes. Takahashi et al. reported that in 22 cases of IgG4-associated salivary gland inflammation (51), the ratio of CD4-positive T lymphocytes to CD8-positive T lymphocytes was 1/2, and the majority of CD8-positive cytotoxic T lymphocytes were found in the periductal and perivacuolar areas.

#### 3.3.2 IgG4-MD

IgG4-MD, also known as Mikulicz syndrome, was first reported as early as 1892. This condition predominantly affects middle-aged and elderly individuals, with an average age of onset at 55 years. It is more common in males in Western countries, while females are predominantly affected in Japan (52).

Clinically, IgG4-MD is characterized by painless, symmetrical, and persistent enlargement of the unilateral or bilateral submandibular, parotid, or lacrimal glands for more than three months. Mild dry mouth and dry eye symptoms may accompany the glandular enlargement, making it easily confused with Sjögren’s syndrome (SS).

While both IgG4-associated Mikulicz’s disease (MD) and Sjögren’s syndrome share common features of glandular enlargement accompanied by lymphocytic infiltration, it’s crucial to recognize the substantial clinical and pathological distinctions between these two conditions (52). On imaging studies such as CT and MRI, specific characteristics can help differentiate between them. In CT scans, IgG4-MD lesions typically exhibit homogeneous hypodensities and uniform enhancement. On MRI, the presence of fibrosis often results in relatively low signal intensity on T2-weighted images (T2WI), while homogeneous enhancement can be observed on T1-weighted images (T1WI) following contrast administration (53).

Sjögren’s syndrome is characterized by prominent symptoms of dry mouth and dry eyes, with relatively less significant glandular enlargement and notable reduction in glandular secretory function. Antinuclear antibodies and anti-SSA/SSB antibodies are frequently detected (54). To differentiate between the two conditions, the Japanese Sjögren’s Syndrome Association proposed diagnostic criteria for MD in 2008: (1) persistent symmetrical enlargement of at least two pairs of glands, including the lacrimal, parotid, and submandibular glands, for more than three months; (2) elevated serum IgG4 levels (≥135 mg/dL); (3) histopathological features of lymphocyte and IgG4+ plasma cell infiltration, with the IgG4+/IgG+ plasma cell ratio exceeding 50%, along with typical tissue fibrosis or sclerosis. The diagnosis requires the fulfillment of either (1) + (2) or (3). Recently, Tsuboi et al. used DNA microarray analysis to investigate labial gland tissue from patients with IgG4-related diseases and SS (51). They discovered distinct gene expression patterns between these two diseases, suggesting different pathogenic mechanisms. Therefore, it was concluded that IgG4-related diseases and SS are not the same disorder (55).

### 3.4 IgG4 related associated autoimmune hepatitis

In 2007, Umemura et al. reported the first case of IgG4-related autoimmune hepatitis (IgG4-AIH) (56), which exhibited biochemical and histological features consistent with the diagnostic criteria proposed by the International Autoimmune Hepatitis Group (IAIHG) for autoimmune hepatitis (AIH). However, this case presented with atypical characteristics compared to classical AIH, as it showed abundant infiltration of IgG4-positive plasma cells in the portal vein and a significant increase in serum IgG4 levels (57). Building upon this enhanced IgG4 antibody response, Umemura proposed a novel disease entity termed IgG4-AIH (58).

IgG4-AIH represents a subtype of AIH rather than an involved site of IgG4-RD. Similar situations have been observed in other IgG4-RDs, particularly in patients with autoimmune pancreatitis (AIP) where infiltration of IgG4-positive plasma cells is commonly found during liver biopsies, but can only be diagnosed as liver damage caused by IgG4-RD. Studies on a small number of patients have suggested that IgG4-AIH and non-IgG4-related AIH share similarities in laboratory findings and histopathological manifestations, with both demonstrating a certain degree of responsiveness to glucocorticoid therapy. However, IgG4-AIH patients may require a shorter duration for alanine aminotransferase normalization after glucocorticoid treatment compared to non-IgG4-related AIH (59). It is important to note that the aggregation of IgG4-expressing plasma cells can only be detected in IgG4-AIH (60). Pathologically, liver tissues of IgG4-AIH patients typically exhibit giant cell formation and “rosy” changes, while the bile ducts are generally unaffected. As a result, the extent of liver damage in IgG4-AIH is often more severe compared to AIP-associated liver damage (58).

Currently, there is a lack of unified diagnostic criteria for IgG4-AIH, necessitating comprehensive consideration of clinical, biochemical, immunological, and liver histopathological examination results. Umemura et al. recommended including the following diagnostic criteria for IgG4-AIH: (1) fulfillment of the diagnostic criteria for AIH; (2) serum IgG4 ≥1.35 g/L; and (3) liver biopsy demonstrating >10 IgG4-positive plasma cells per high-power field in the portal area. Some researchers also propose that the ratio of IgG4-positive plasma cells to IgG1-positive plasma cells >1 in liver tissue can serve as one of the diagnostic criteria for this disease. Chung et al. considered a cut-off value of ≥5 IgG4-positive plasma cells per high-power field in the liver tissue as the diagnostic criterion for IgG4-AIH (61).

### 3.5 IgG4-related gastropathy

A study by Shinji et al. in 2004 found that patients with AIP have a significantly higher incidence of gastric ulcer compared to the control group (62). Histological examination of gastric tissues revealed a prominent infiltration of IgG4-positive plasma cells, suggesting the gastric involvement of IgG4-RD. This led to the proposal of a new concept known as AIP-G.

AIP-G is a subtype of AIP characterized by chronic gastritis. Endoscopic examination commonly shows gastric ulcers and chronic inflammation, with negative detection of Helicobacter pylori. Biopsy samples from the mucosal layer demonstrate abundant infiltration of plasma cells, lymphocytes, and eosinophils, particularly IgG4-positive plasma cells. Neutrophil infiltration, on the other hand, is rarely observed (63). Additionally, AIP-G patients exhibit impaired gastric emptying function, which may be attributed to extensive lymphoplasmacytic infiltration and fibrosis in the gastric mucosa. Furthermore, the compromised exocrine function in AIP patients can also impact gastric emptying speed (62).

Uehara et al. summarized 39 cases of gastric involvement in IgG4-RD patients, of which only 40% presented typical gastric manifestations (63). The majority of gastric lesions observed in these patients were characterized as inflammatory tumors, ulcers, nodular lesions, chronic gastritis regions, and malignant conditions. A meta-analysis indicated a relative risk of 1.69 for the development of gastric cancer in IgG4-RD patients (64).

### 3.6 IgG4-related sclerosing mesenteritis

IgG4-related disease involving the mesentery primarily presents as sclerosing mesenteritis, a rare chronic condition characterized by localized or diffuse fibrosis and inflammation of the mesentery in the small intestine. Patients affected by this condition typically exhibit nonspecific abdominal symptoms such as pain, bloating, diarrhea, or even ischemia. Clinical management of sclerosing mesenteritis can be quite challenging (65). On imaging studies, sclerosing mesenteritis appears as a soft-tissue mass enveloping the mesenteric vasculature. This radiological appearance can be similar to other mesenteric diseases like lymphomas, carcinoid tumors, and carcinomas. Distinguishing sclerosing mesenteritis from retroperitoneal fibrosis can be particularly challenging. However, certain features on CT scans, such as the presence of an “epimastigote-like mesentery” and the observation of the “fat ring sign” on the wall of the mesenteric vasculature, can be indicative of sclerosing mesenteritis.

### 3.7 Ormond’s disease

Ormond’s Disease, also known as IgG4-related retroperitoneal fibrosis, is a form of IgG4-related disease primarily affecting the retroperitoneum. It is characterized by idiopathic fibrosis of the retroperitoneum and belongs to a group of chronic periaortitis disorders, including IgG4-related peritoneal fibrosis, periaortitis, and inflammatory aortic aneurysm. The typical clinical manifestations include vague pain localized in the back, flank, lower abdomen, and inner thigh, as well as lower limb edema and hydronephrosis (66). It is important to note that the mild nature of the symptoms often leads to misdiagnosis. In imaging studies, IgG4-associated retroperitoneal fibrosis typically presents as a soft tissue mass situated in the retroperitoneal region, enveloping the abdominal aorta and its branching vessels. In some cases, it can encircle the ureter, potentially resulting in pyeloureteral hydrops. Additionally, periaortic lesions, known as IgG4-associated periaortic inflammation, appear as non-stenotic masses with irregular margins. These lesions exhibit uniform delayed enhancement on CT scans and may be associated with aortic dilatation (67).

## 4 Treatment of IgG4-DSD

Treatment of IgG4-RD and IgG4-DSD follows similar approaches. In 2015, a consensus statement on the management and treatment of IgG4-RD was published (13).This statement was developed through collaborative efforts of experts from various medical specialties and countries. Timely diagnosis and treatment are of utmost importance to slow disease progression, preserve organ function, and prevent irreversible damage caused by chronic inflammation and fibrosis. Therefore, it is recommended to initiate treatment early for any symptomatic and active IgG4-RD patient. Currently, the fundamental treatment strategies include glucocorticoids, conventional disease-modifying anti-rheumatic drugs (DMARDs), B-cell depletion therapy, and surgical interventions.

### 4.1 Glucocorticoids

Glucocorticoids are the first-line therapy for the treatment of IgG4-DSD. Currently, the international consensus advocates for an initial treatment regimen of oral prednisone at a dose of 30-40 mg/day or 0.6 mg/(kg·day). After 2-4 weeks of treatment, the dose is gradually reduced by 5 mg every 1-2 weeks based on clinical manifestations, radiological findings, and laboratory results. The aim is to taper the dose to 5 mg/day over a period of 3-6 months and discontinue the medication (13). Japanese expert consensus recommends long-term maintenance therapy with 2.5-5.0 mg/d prednisolone for patients at high risk of recurrence (68).

Although initial remission rates are high after short-term immunization shocks with glucocorticosteroids, outbreaks and relapses are still common (69). Therefore, despite the risk of osteoporosis, diabetes mellitus, and infection associated with long-term steroid use, long-term use of 5 mg/d for at least 3 years is recommended (70).

### 4.2 Conventional disease-modifying anti-rheumatic drugs

DMARDs are used as adjunctive therapy to glucocorticoids in the treatment of IgG4-DSD (71). The purpose of their use is to reduce the toxicity of glucocorticoids, decrease the recurrence rate of the disease, or minimize the dosage of steroids. Common regimens include azathioprine, methotrexate, hydroxychloroquine, and tacrolimus (see **Table 4**), and data on these agents are limited to retrospective analyses, case series, or case reports and are insufficient to guide clinical decision making. Prospective studies have also confirmed the high failure rate of treatment regimens combining glucocorticoids and DMARDs (82). Thus the use of DMARDs in IgG4-DSD remains controversial.

**Table 4:** List of DMARDs in IgG4-DSD Treatment.

Drug	Initial dose	Maintenance dose	Research type	Result	Ref.
Mycophenolate Mofetil	1-2 g/d	0.5-1 g/d	Randomized unblinded control	DR: 76.47%	(72)
Azathioprine	2-2.5 mg/kg/d	<2 mg/kg/d	Meta-analysis	DR: 80.8%	(73)
Amethopterin	10-20 mg/week	15-20mg/week	Case report	DR: 60%, PR: 40%	(74, 75)
Leflunomide	20 mg/d	20mg/d	Randomized unblinded control	RR: 18.2 %	(76)
Cyclophosphamide	50-100 mg/d (3 months)	50 mg/d	Prospective cohort study	DR: 88%	(77)
Cyclosporin a	150 mg/d	150 mg/d	Case report	–	(78)
Tacrolimus	1-2.5 mg/d	1-2.5 mg/d	Chart review	RR: 20%	(79)
Iguratimod	25 mg, *bid*	25mg, *bid*	Prospective cohort study	CR:30%, PR:56.7%	(80)
Thalidomide	50 mg/d	100 mg/d	Case report	–	(81)

DR, disease remission; PR, partial remission; CR, complete remission; RR, recurrence rate.

–, not reported.

### 4.3 B cell-targeted therapy

B-cell targeted therapy represents a precision clearance approach rooted in the elevated presence of B lymphocytes and CD4 T cells during the pathological progression of IgG4-related diseases. The underlying mechanism of action hinges on the depletion of B cells, which serves multifaceted purposes. This strategy effectively impedes the presentation of antigens to CD4 cytotoxic T lymphocytes, concurrently mitigating tissue fibrosis, and lowering serum IgG4 concentrations. By diminishing the production of immune complexes, this therapy safeguards tissues from damage, marking a pivotal breakthrough in recent research endeavors. In recent years, therapeutic interventions centered on B-cell targeting have garnered substantial attention and enthusiasm among the scientific community (83).

Rituximab (RTX) stands out as the earliest and most extensively researched B-cell targeted therapy, demonstrating unequivocal efficacy (84). This drug serves as the quintessential representative of anti-CD20 monoclonal antibodies. As far back as 2010, RTX emerged as a promising treatment for IgG4-related diseases. Its specificity lies in binding to CD20 antigens that adorn the surfaces of precursor B cells, mature B cells, and memory B cells. Through various mechanisms, including antibody-dependent cell-mediated cytotoxicity, antibody-independent cell phagocytosis, and complement-independent cytotoxicity, RTX orchestrates the elimination of B lymphocytes (85).Notably, RTX assumes a crucial role in long-term maintenance therapy, particularly for younger patients grappling with the challenging aspects of the disease. This drug emerges as a lifeline for individuals with an early onset of the disease who struggle with adverse effects, delivering outstanding clinical efficacy in these cases (86).

Except for RTX, XmAb5871 represents a monoclonal antibody targeting FcγRIIb and CD19, effectively suppressing various facets of B-cell functionality, including activation, proliferation, and antigen presentation, employing multiple pathways (87). In a notable phase 2 trial, this therapeutic approach yielded a positive response in 2 out of 15 patients, with the majority achieving disease control without the need for glucocorticoids (88). On another front, Inebilizumab (89), a humanized monoclonal antibody directed against CD19, operates by eliminating B-cell populations expressing CD19 through antibody-dependent cell-mediated cytotoxicity. Furthermore, the realm of treatment for IgG4-related diseases is expanding to include Bruton’s tyrosine kinase inhibitors (BTKi). These inhibitors play a pivotal role in regulating B-cell proliferation and differentiation by thwarting signaling pathway transduction mediated by B-cell receptor (BCR) activation. Two BTK inhibitors, namely rilzabrutinib (PRN1008) and zanubrutinib, are currently under evaluation in randomized clinical trials, offering promising prospects for future therapeutic options. Remarkably, bortezomib, a proteasome inhibitor, has showcased success in treating IgG4-RD. This innovative approach has been validated, with case reports highlighting its efficacy, particularly in patients grappling with IgG4-RD lung involvement combined with periorbital inflammatory pseudotumor (90).

### 4.4 T cell-targeted therapy

The pathogenesis of IgG4-related disease involves various types of T cells, including helper T cells 2 (Th2), regulatory T cells (Treg), and follicular helper T lymphocytes (Tfh), all of which play crucial roles in the disease progression. Consequently, targeting T cells has emerged as a promising therapeutic strategy for IgG4-RD, albeit with limited current treatment options. Abatacept is a recombinant fusion protein with its mechanism of action involving cytotoxic T lymphocyte-associated protein 4 (CTLA-4). It interferes with antigen presentation by competitively binding to CD80/86 on the surface of antigen-presenting cells, thereby inhibiting the activation of T cells. This mechanism holds potential for application in the treatment of IgG4-RD. Elotuzumab, on the other hand, is a monoclonal antibody that targets SLAMF7 (also known as CS1) protein. It operates by selectively inhibiting CD4+ cytotoxic T lymphocytes (CTLs) expressing SLAMF7, aiding in the control of the pathogenic process in IgG4-RD (83). Recent reports indicate the effectiveness of elotuzumab in the treatment of IgG4-related sclerosing mesenteritis (91).

### 4.5 Surgical treatment

Biliary drainage is a necessary adjunctive treatment for patients with severe cholestasis or pressure symptoms caused by other organ involvement; surgery is also required for malignant tumors due to space-occupying lesions. However, the pros and cons of surgery need to be fully weighed, and there are many reports that surgery does not bring more benefit to the disease, or even does not alleviate the disease (92).

## 5 Conclusion

Recent years have witnessed an increasing recognition of IgG4-RD, an immune-mediated condition characterized by end-stage fibrosis that can lead to multi-organ failure and significantly impact patients’ quality of life. However, the lack of specific diagnostic and therapeutic tools often results in missed or misdiagnosed cases, leading to delayed treatment initiation. Notably, IgG4-DSD has emerged as the most prevalent subtype among IgG4-RD, prompting increasing attention from clinicians.

Despite advancements in understanding IgG4-DSD, its pathogenesis remains incompletely elucidated, and there is a lack of standardized diagnostic criteria. Furthermore, the pros and cons of various treatment options have not been definitively established, and patients often experience high recurrence rates. Clinical studies have indicated that elevated serum IgG4 levels serve as a useful diagnostic marker, alongside imaging findings. However, the main challenge lies in differentiating IgG4-DSD from malignant tumors, with pathological diagnosis being considered the current “gold standard.” Moreover, the effective treatment of glucocorticoid can also be used as a reference.

Controversies persist regarding the management of IgG4-DSD, with conventional interventions being commonly employed but numerous research questions remaining unanswered. Hence, healthcare providers should enhance their understanding of IgG4-DSD and undertake further research to explore its underlying mechanisms and clinical characteristics comprehensively. These endeavors aim to advance the diagnosis and treatment of this condition, and improve patient outcomes.

## Author contributions

SW: Writing – original draft. HW: Writing – review & editing.
